# First Principle Study of the Mechanical Properties and Phonon Dispersion of the Iron Pnictide Compound EuFe_2_As_2_

**DOI:** 10.1155/2020/5986073

**Published:** 2020-09-26

**Authors:** N. K. Omboga, C. O. Otieno, P. W. O. Nyawere

**Affiliations:** ^1^Department of Physics, Kisii University, P.O. Box 408, Kisii, Kenya; ^2^Department of Physical and Biological Sciences, Kabarak University, P.O. Box Private Bag Kabarak 20157, Nakuru, Kenya

## Abstract

We present results on the first principle study of the elastic constants and the phonon dispersion of EuFe_2_As_2_ at zero pressure. The ground-state energy calculations were performed within Density Functional Theory (DFT) and the generalized gradient approximation using the pseudopotential method with plane-wave basis sets. The projector augmented-wave (PAW) pseudopotentials were used in our calculation. The open source code QUANTUM ESPRESSSO was used with its pseudopotential database. The study on the elastic constants at zero pressure was a clear indication that the compound is mechanically stable, and the phonon dispersion study also indicated that the compound is dynamically stable. The elastic constants and mechanical properties also led to the conclusion that the compound is ductile and anisotropic.

## 1. Introduction

Iron pnictides belong to the “122” family of compounds and have in studies of the recent past shown to exhibit high temperature superconductivity [1–4]. These are basically type II superconductors and they consist of iron arsenide layers. By substitution of europium with sodium or potassium, the material becomes superconducting. Also, in the application of external pressure or doping, superconductivity is achieved [4]. There is a reduction in phase transition temperature when the compound is doped or adequate pressure is applied on it, making it superconducting [5].

The iron pnictide compounds are also unique because their phase transition to superconductivity and magnetic properties are connected to structural properties and conceal the key to comprehending the basic properties of these materials [6]. According to [7], EuFe_2_As_2_ experiences a phase transition from the tetragonal phase to orthorhombic phase at a pressure of 4.3 GPa, which is maintained until a pressure of 11 GPa where it transits to a collapsed tetragonal phase. This pressure is maintained up to around 35 GPa. The conductivity of iron pnictides shows a highly anisotropic characteristic [8].

Phase transition at low temperature is common in the iron pnictide family of compounds. Experiments performed at high pressure reveal characteristics which link the electronic and structural properties to the superconducting properties of the compound [6].

Superconductivity and ferromagnetism coexist in europium diiron diarsenide doped with phosphorous [5]. The iron spin density and the europium (Eu^2+^) antiferromagnetically make europium diiron diarsenide outstanding among the 122 iron pnictide superconducting compounds. The FeAs layers in the compound europium diiron diarsenide are seen to be the source of the superconductivity [4]. This compound is also unique because of the additional magnetic moment of the localized Eu^2+^ given that other 122 compounds undergo only the SDW transition.

Application of hydrostatic pressure on EuFe_2_As_2_ indicates that the SDW transition is continuously suppressed on pressure application. Superconductivity is achieved on suppression of the magnetism. Superconductivity transition temperature occurs at 30 K at a pressure of 2.8 GPa [9].

Other studies in similar iron pnictides have been done and documented, although mostly experimental. Barium diiron diarsenide exhibits superconductivity on distorting the structure through application of pressure [10]. Superconductivity is achieved at a pressure of 5.5 GPa and temperature of 30.5 K. Studies on CaFe_2_As_2_ have also revealed that it exhibits superconductivity on application of pressure and up to temperatures of 40 K [11]. Experimental results on europium diiron diarsenide have also indicated that superconductivity occurs below temperatures of 33 K [12].

In the ground state, pnictides show spin density wave. The main differentiating factor between Cuprates (which were initially the major materials under the study of superconductivity) is the behavior of the electrons that conduct [13]. In iron pnictides, the Fe 3*d* prevails over the electrons near the Fermi level. The *p* character is largely contained in the states near the Fermi level [13]. EuFe_2_As_2_ has unique characteristics, since the Eu^2+^ ions have 4*f* electrons and the combined spin of the electrons is 7/2 [14]. In this article, we present the elastic constants and phonon dispersion of the iron pnictide EuFe_2_As_2_ as studied from first principles. Seismic wave velocities can be explained using elastic constants and also the comprehending of chemical bonds is facilitated by the elastic constants [15]. This paper is arranged such that Section 2 presents computational methodology, Section 3 reports on results and discussions, and Section 4 is conclusion.

## 2. Computational Details

The open source code QUANTUM ESPRESSO was used in this study. Projector augmented-wave (PAW) pseudopotentials of the Perdew, Erzenhoff, and Burke (PBE) were employed to cater for the core electrons [16]. The generalized gradient approximation (GGA) [17] functional was employed for the exchange correlation. The Kohn–Sham equations were solved iteratively by running a self-consistent cycle. The unit cell used to generate the super cells comprised 5 atoms: iron and arsenic had two atoms each and europium had one atom. Nine super cells of 2 × 2× 2 dimensions were created for the phonon dispersion study, and the number of atoms in each generated super cell was 40. The phonon calculation of finite differences was done using Phonopy code. A smearing of 0.2 eV of the Methfessel-Paxton scheme was used. We used an energy cut-off of 30 Ry, which dictated the number of plane waves to be employed. The elastic constants were obtained using the post-processing code THERMO_PW within Quantum ESPRESSO. Variable cell relaxation was done before undertaking all the above calculations to ensure the material is stress-free.

Bulk modulus is a measure of the ability of a material to withstand variations in volume when compressed on all sides [18]. The bulk modulus in this study was computed using

(1) $$\begin{array}{c} B_{V}=\frac{2C_{11}+2C_{12}+C_{33}+4C_{13}}{9},\\ B_{R}=\frac{C^{2}}{M}, \end{array}$$

where *C*^2^=(*C*_11_+*C*_12_)*C*_33_ − 2*C*_13_^2^.

And, *M*=*C*_11_+*C*_12_+2*C*_33_ − 4*C*_13_.

Then, *B*=*B*_*V*_+*B*_*R*_/2.

The shear modulus (*G*) is the measure of resistance to reversible deformations upon shear stress [18].

For the shear modulus,

(2) $$\begin{array}{c} G_{V}=\frac{M+3C_{11}-3C_{12}+12C_{44}+6C_{66}}{30}. \end{array}$$

Therefore, *G*=*G*_*V*_+*G*_*R*_/2.

The Young's modulus *E* [18] and Poisson's ratio *V* [18] were obtained, respectively, using the equations below:

(3) $$\begin{array}{c} E=\frac{9GB}{3B+G},\\ V=\frac{3B-2G}{2\left(3B+G\right)}. \end{array}$$

The study of phonons assists in understanding properties of materials such as thermal and electrical conductivity. It enables quantization of the energy of vibration. A phonon refers to a kind of lattice vibration in a crystal, where the particles vibrate at the same single frequency [19]. From the phonon dispersion calculation, one can be able to calculate the critical temperature of the material in question. A dispersion relation refers to the relationship between the frequency of vibration and the wave vector and this relationship is given as follows:

(4) $$\begin{array}{c} \omega =v\left(k\right), \end{array}$$

where *k* is wave vector, *ω* is frequency of vibration, and *v* is velocity of sound [19, 20].

## 3. Results and Discussion

Structural optimization was done by doing a variable cell relaxation calculation and also optimizing K-points and cut-off energy. The lattice parameters of the tetragonal crystal structure were also calculated being guided by parameters from previous experimental studies on the compound and optimized to obtain a relaxed structure, [21], and the parameters are as shown in Table 1.

From Table 1, it is realized that optimized and experimental lattice parameters are in good agreement [22].

**Figure 1:**
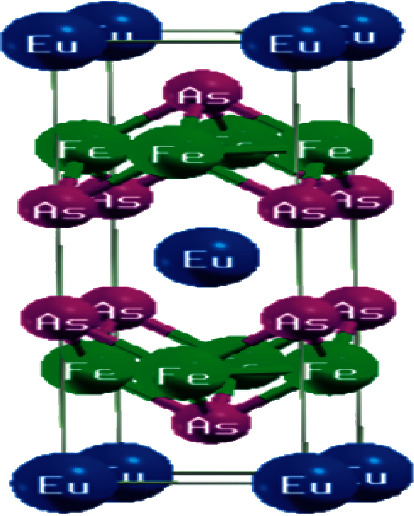
The crystal structure of EuFe_2_As_2._ The structure was viewed using Xcrysden, a package in QUANTUM ESPRESSO. The structure is made up of three atoms, namely, europium, iron, and arsenic. It is similar to the structure in past studies on the compound as seen in [23].

The crystal structure of this iron pnictide compound EuFe_2_As_2_ is as shown in Figure 1:

Mechanical properties such as elastic properties guide in understanding more about the nature of the force in the crystal. The elastic constants c_ij_ of the compound were computed at *T* = 0 K and *P* = 0 GPa. There exist six elastic constants in the body centered tetragonal structure [22], and they are computed and reported for the first time in Table 2.

Given the Born-Huang criteria [24] shown below, a stable tetragonal structure should satisfy the following criteria:

- *C*_*ii*_ > 0 (*i* = 1, 3, 4, 6)
- *C*_11_ + *C*_33_ − 2*C*_13_ > 0
- 2 (*C*_11_ + *C*_12_) + *C*_33_ + 4*C*_13_ > 0
- *C*_11_ − *C*_12_ > 0

The values reported in Table 2 are all positive and satisfy all the above four criteria, hence proving that this tetragonal compound is stable. *C*_11_ > *C*_33_ as seen from Table 2, implying that the crystal is more stiff along 100 and 010 axes, results which are in agreement with the study on a similar iron pnictide BaPd_2_As_2_ [25]. *C*_12_–*C*_44_ gives the Cauchy pressure, which, if positive, makes the compound ductile. From Table 2, the Cauchy pressure is positive; hence the material is ductile, a result similar to that of a related iron pnictide [25].

In our earlier studies, we reported the bulk, shear, and Young's modulus, and Poisson's ratio values of EuFe_2_As_2_ at zero pressure; we tabulate these earlier results here for completeness [21] in Table 3. Bulk, shear, and Young's modulus are obtained using the Voigt–Reuss–Hill approximation method.

Pugh's ratio, *B*/*G* [26] indicates the following:

*B*/*G* >  1.75 for ductile materials and *B*/*G* >  1.75 for brittle materials.

The *B*/*G* value from Table 3 gives a value of 2.53, implying that the compound is ductile. The value of Pugh's ratio for this compound is similar to that of BaPd_2_As_2_, an iron pnictide of similar structure [25].

Using the Frantserich method, ductile and brittle materials are classified on the basis of their Poisson's ratio. The value *v* ~ 0.26 acts as the border of ductility and brittleness.

If *v* > 0.26, then the material is ductile and if *v* > 0.26, the material is brittle. Since *v* > 0.26, as seen from Table 3, this is further proof that the compound is indeed ductile. Also, Poisson's ratio is in close range with that obtained in the study of the iron pnictide BaPd_2_As_2_ [25]. Poisson's ratio also suggests that the compound is metallic, which is in good agreement with a similar pnictide in [25].

When Poisson's ratio is in the range 0.25–0.5, it implies that the forces present in the compound are central [25]. The anisotropic character was also calculated using

(5) $$\begin{array}{c} A^{U}=\frac{5G_{V}}{G_{R}}+\frac{B_{V}}{B_{R}}-6, \end{array}$$

where if *A*^*U*^ = 0, the compound is totally isotropic.

In this case, the calculations indicate that the material is anisotropic since the value is 0.408. This is in good agreement with past studies of compounds in the pnictide family that have also been proved to be anisotropic as in [25]. The bulk, shear, and Young's moduli values increased upon application of increasing pressure on the compound. For instance, Poisson's ratio, bulk modulus, shear modulus, and Young's modulus at a pressure of 1 GPa were 0.36100, 1196.9 GPa, 326.6 GPa, and 889.0 GPa, respectively, showing a progressive increase up to 1 GPa.

**Figure 2:**
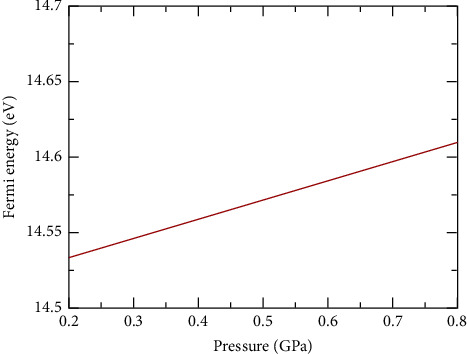
A graph of pressure against the Fermi energy from 0.2–0.8 GPa. The Fermi energy increases with an increase in pressure up to 0.8 GPa.

The Fermi energy of increasing pressure up to 0.8 GPa was obtained and plotted, and the result is as seen in Figure 2.

Figure 2 indicates that the population of charge carries with respect to density of states increases; hence, more electrons are made available for electrical conductivity, enhancing electrical conductivity in the compound. The Fermi energy also is important in describing the electronic structure properties of a material.

**Figure 3:**
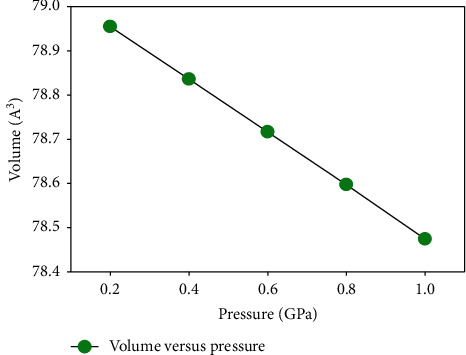
Up to a pressure of 1 GPa, application of pressure on the system leads to a decrease in the volume as expected from past experimental studies on the pnictide [1, 11, 17].

The change in volume of the crystal upon application of pressure was also obtained, and a graph of volume against pressure was plotted as seen in Figure 3.

## 4. Phonon Dispersion

**Figure 4:**
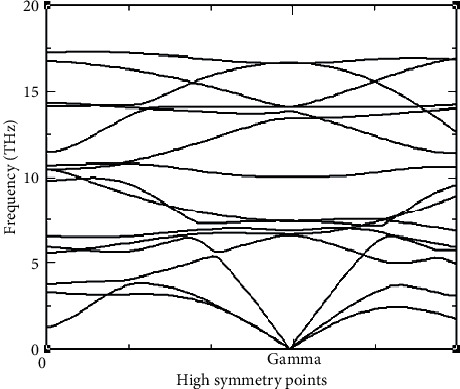
The phonon dispersion curve. The optical (upper) mode and the acoustic (lower) mode are clearly differentiated. Given that europium diiron diarsenide has got five atoms, there are 15 modes of vibration. The acoustic modes converge at the gamma high symmetry point as shown in Figure 4.

There are two branches in phonon dispersion relation: the acoustic mode which is the lower mode and the optical mode which is the upper mode [27]. The acoustic mode refers to the in-phase vibration mode while the optical mode refers to the out-of-phase vibration. The optical phonons get their name from the fact that they get excited by the radiation of infrared in crystals that are ionic [28]. There are two types of phonon calculation methods: the frozen phonon method and the Density Functional Perturbation Theory [29, 30] as implemented in PWSCF.  Figure 4 shows the phonon dispersion for the iron pnictide from the current study.

In the study of phonons, there exist three modes that are associated with each mode number *n* [19], taking into account the *x*, *y*, and *z* axes [31]. The number of acoustic modes is usually three for crystals whose number of atoms is equal to or greater than two, and the optical modes are given by 3N-3 [28]. Therefore, given that europium diiron diarsenide has got five atoms, there are 15 modes of vibration expected. As seen in Figure 4, there are 15 vibration modes with twelve being optical modes and three being acoustic modes. The acoustic modes converge at the gamma high symmetry point. Acoustic modes vibrate at a slower frequency and are in the same phase with the unit cell. Optical modes of vibration have a higher frequency compared to acoustic modes and two neighboring atoms vibrate in a direction opposite to each other. In the acoustic mode, the two adjacent atoms will vibrate together in the same direction. Phonon dispersions are computed along a given line of high symmetry points. The above information therefore confirms that the compound is dynamically stable.

**Figure 5:**
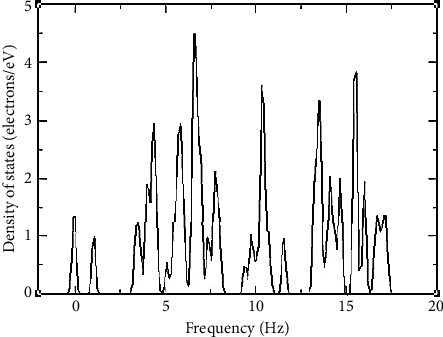
The plotted phonon density of states. As the wavelength becomes longer, the frequency of the acoustic modes of vibration goes to zero, while for longer wavelengths in optical phonons, the frequency does not go to zero.

The phonon density of states was also plotted and it is as shown in Figure 5.

A system is considered to be dynamically stable at equilibrium if the potential energy is always increasing for any combination of displacement of atoms; therefore, phonons should have non-negative and real frequencies for stability [32]. Negative frequencies imply that the potential energy reduces; hence, the system is unstable. Phonon frequencies arise as a result of the displacement of atoms in a given crystal from the rest position, which in turn makes the forces rise [32]. It is important to establish the number of normal modes that are neighboring a certain phonon energy; these details are necessary when studying thermal and electrical conductivity and also establishing the critical temperature of superconducting materials [33]. The Debye temperature is a constant that is associated with the highest allowed mode of vibration [34]. The Debye temperature in this study was 436.454 K and the average Debye sound velocity was 3330.336 m/s. A Debye temperature of above 400 K implies that the crystal's thermal conductivity is high [35], and since the compound we were studying had a Debye temperature of above 400 K, we concluded that the thermal conductivity is high. Note that, for temperatures that are below the Debye temperature, the heat capacity of the compound rises with the temperature cube and for temperatures above the Debye temperature, the heat capacity of the crystal remains constant; that is to say, it no longer depends on temperature. The Debye temperature and the heat capacity are directly proportional [36].

## 5. Conclusion

We presented results on the elastic constants and phonon dispersion of the iron pnictide EuFe_2_As_2_, studied from first principles. For the first time, we have reported elastic constants and Debye temperature of this iron pnictide. From the study, it can be concluded that the compound is mechanically and dynamically stable. The iron pnictide is ductile and anisotropic. The compound is also a good thermal conductor deduced through its Debye temperature.

## Figures and Tables

**Table 1:** Experimental and calculated structural values.

Parameter	This work	Experimental	Reference
*a*_0_ = *b*_0_ (ang)	3.945	3.916	[11]
*c*_0_ (ang)	12.846	12.052	[11]

**Table 2:** Elastic constants.

*C*_*ij*_	Value (GPa)
*C*_11_	531.61
*C*_12_	284.81
*C*_13_	253.34
*C*_33_	505.73
*C*_44_	141.31
*C*_66_	225.69

**Table 3:** Bulk, shear, and Young's modulus, and the Poisson ratio of EuFe_2_As_2_.

Property	Voigt approximation	Reuss approximation	Voigt–Reuss–Hill average
Bulk modulus (*B*)	346.939 GPa	346.352 GPa	346.646 GPa
Young's modulus (*E*)	375.378 GPa	350.224 GPa	362.801 GPa
Shear modulus (*G*)	142.224 GPa	131.518 GPa	136.871 GPa
Poisson ratio (*n*)	0.31967	0.33147	0.3254

## Data Availability

All the data in this study are available and can be made available on request with the corresponding author.
